# Operationalizing systemic multi-hazard and multi-risk assessment: Lessons from the MYRIAD-EU framework

**DOI:** 10.1016/j.isci.2026.114935

**Published:** 2026-02-07

**Authors:** Stefan Hochrainer-Stigler, Robert Šakić Trogrlić, Karina Reiter, Anne Sophie Daloz, David Geurts, Lin Ma, Noemi Padrón-Fumero, Sharon Tatman, Silvia Torresan, Carmen D. Álvarez-Albelo, Veronica Casartelli, Roxana Ciurean, Maria Katherina Dal Barco, Jaime Díaz-Pacheco, Juan José Díaz-Hernández, Pedro Dorta Antequera, Melanie J. Duncan, Davide Mauro Ferrario, Sara García-González, Stefania Gottardo, Raúl Hernández-Martín, Abel López-Díez, David Romero Manrique, Diep Ngoc Nguyen, Marleen C. de Ruiter, Nikita Strelkovskii, Philip J. Ward

**Affiliations:** 1 Advancing Systems Analysis Programme, International Institute for Applied System Analysis, 2361 Laxenburg, Austria; 2 Center for International Climate Research (CICERO), 0349 Oslo, Norway; 3 Deltares, 2629 HV Delft, the Netherlands; 4 University of La Laguna, La Laguna, 38200 Santa Cruz de Tenerife, Spain; 5 Venice Ca’ Foscari University, 30175 Venice, Italy; 6 CMCC Foundation - Euro-Mediterranean Center on Climate Change, 30175 Venice, Italy; 7 British Geological Survey, NG12 5GG Keyworth, UK; 8 Institute for Environmental Studies (IVM), Vrije Universiteit Amsterdam, 1081 HV Amsterdam, the Netherlands

**Keywords:** Earth sciences, Environmental science, Environmental issues

## Abstract

Multi-hazard and multi-risk contexts are increasingly recognized as central to disaster risk reduction and climate adaptation. While there is a recognized need to move beyond single-hazard and single-sector approaches, practical frameworks for systemic multi-hazard and multi-risk assessment remain scarce. In response, the Horizon 2020 Multi-hazard and sYstemic framework for enhancing Risk-Informed mAnagement and Decision-making in the E.U. (MYRIAD-EU) project developed a conceptual framework grounded in systemic risk research and structured around a six-step iterative process. This paper critically reflects on its implementation across five European pilot regions. Using project deliverables, a survey, and a focus group, we assess the framework’s strengths and limitations and distil lessons learned from both its development and its practical application. These lessons learned are that the framework provides a valuable roadmap for structuring complexity, fostering dialogue with stakeholders, and distinguishing direct from indirect risks. However, challenges remain regarding data, capacity, tool integration, and communication. We conclude with recommendations for improving usability, institutionalization, and long-term uptake.

## Graphical abstract

**Figure:**
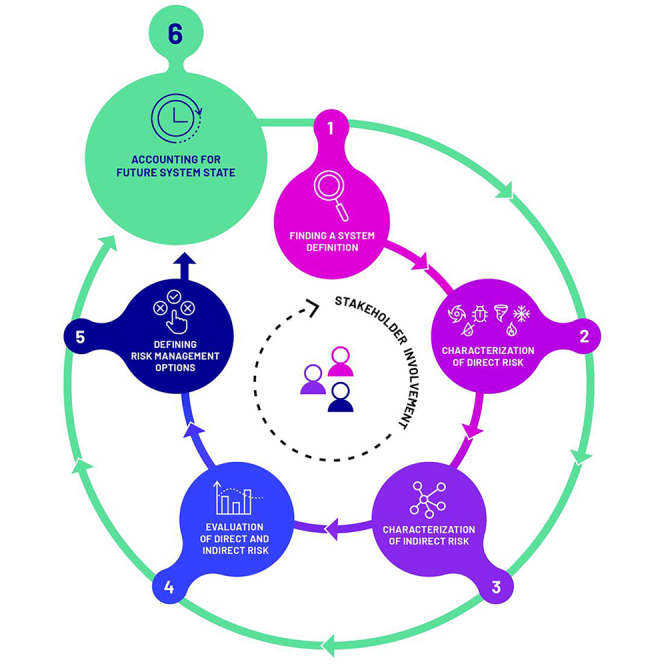

## Introduction

Multi-hazards and their resulting multi-hazard risks are gaining unprecedented traction in disaster risk reduction (DRR), not only because of advances in science and policy but also due to the increasing frequency, severity, and global impact of multi-hazard events.^1^^,^^2^ These trends are increasingly attributed to climate change, which is intensifying compound and cascading hazards and amplifying systemic risk, thereby heightening the urgency for effective and anticipatory climate change adaptation decision-making.^3^^,^^4^^,^^5^^,^^6^ This growing awareness underscores a heightened need for coordinated initiatives such as the Multi-hazard and sYstemic framework for enhancing Risk-Informed mAnagement and Decision-making in the E.U. (MYRIAD-EU) project, which aim to operationalize systemic, cross-sector multi-hazard risk assessment. MYRIAD-EU is part of a new generation of high-level Horizon projects addressing multi-hazard and multi-risk challenges across sectors—such as DIRECTED, PARATUS, REST-COAST, and AGORA—yet it distinguishes itself through its explicit system-of-systems perspective, strong emphasis on operational co-production, and real-world testing of its conceptual framework across five diverse pilot regions. Additionally, the need for multi-hazard risk assessment is explicitly stated in the Sendai Framework for Disaster Risk Reduction 2015–2030,^7^ and the first European Climate Risk Assessment (EUCRA) indicates a need to better characterize compound and cascading risks.^8^ There is emerging evidence on the urgency of moving from single-hazard and single-sector-based approaches to multi-hazard and multi-sector risk assessment and management.^9^^,^^10^

While there have been considerable developments in understanding and characterizing multi-hazards risks, a need for a harmonized framework to guide multi-hazard risk assessment and management across scales and different contextual settings was previously identified.^11^ In response to this need, and as part of the MYRIAD-EU project, a novel conceptual framework for systemic multi-hazard and multi-risk assessment and management was proposed by Hochrainer-Stigler et al.^12^ in a perspective piece in iScience. This conceptual framework was informed by the state of the art in multi-hazard risk science. It is based on systemic risk research ideas, especially in terms of its focus on the identification of clear system boundaries, interconnectedness between elements of the systems, and a system-of-systems approach.

**Figure 1:**
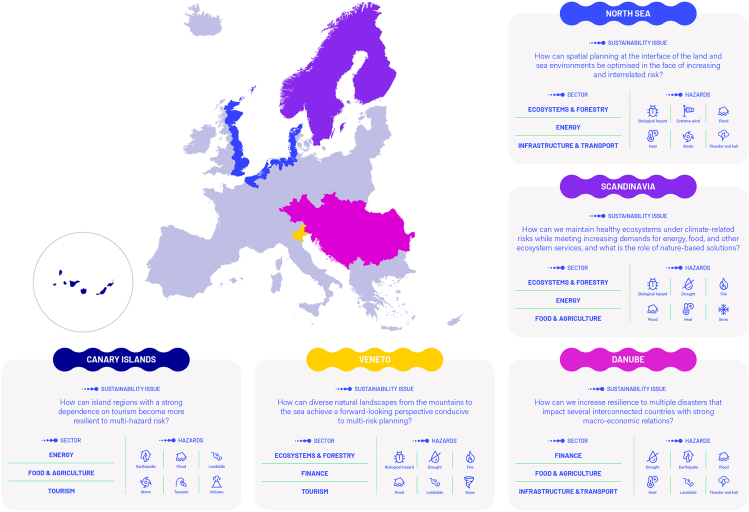
Location of pilots, including representation of their unique sustainability issues, consideration of natural hazards, and sectors of interest where the framework was implemented (adopted from Šakić Trogrlić et al.^13^)

In this paper, we present a critical reflection on the real-world testing of the framework, based on its application within five pilot case study areas in Europe, namely, Scandinavia, the Danube Region, the Veneto Region, the North Sea, and the Canary Islands (Figure 1). Over the last 4 years, the pilot studies have implemented the framework using a vast array of methodological tools and through co-production with stakeholders (an overview of the stakeholder engagement approach used is detailed in Šakić Trogrlić et al.^13^). Stakeholder engagement involved two focus group discussions (FGDs) with core stakeholders and two workshops with a broader stakeholder base. For instance, in the Danube Pilot, focus group 1 (FG1) centered on engaging stakeholders to gather feedback on interconnectedness, agent-based modeling (ABM) outputs, and the application of the Dynamic Adaptive Policy Pathways (DAPP)- Multi-Risk (MR) framework^14^ for scenario development and sectoral pathway building. Meanwhile, FG2 focused on reviewing preliminary ABM results for the Danube Region, discussing sector-specific impacts of hazards (floods, earthquakes, droughts), exploring cross-sectoral and cross-border policy implications, and identifying how modeling outputs can support practical disaster risk management and climate adaptation strategies. This process aimed to align the analysis with stakeholder priorities, contextualize the selection of tools and methods, and validate the findings. Each of the pilot case study areas presents a unique empirical case, experiencing various combinations of multi-hazards, different risk contexts, and differing problems in relation to multi-hazard risks (e.g., economic tourism dependence in the Canary Islands, spillover impacts of multi-hazards in the Danube Region) (Figure 1). We critically reflect on the benefits and limitations of the proposed framework, based on a structured analysis of project deliverables, an open-ended survey, and an FG with research teams in the five pilot regions.

The framework and the tools developed within MYRIAD-EU provide concrete value for multiple audiences: (1) for the pilot case studies, they offer structured guidance for navigating complex, interconnected risk landscapes and tailoring methodological choices to local priorities; (2) for the DRR research community, they contribute a harmonized and empirically tested foundation for advancing systemic multi-hazard risk science; and (3) for DRR practitioners, they deliver actionable, co-produced approaches that support more integrated, cross-sectoral decision-making in practice.

The article is structured as follows: Section two - a short recap of the conceptual framework - gives a short overview of the framework and its main characteristics. Section three - critical reflections on the framework application in MYRIAD-EU pilots - provides a detailed analysis of the three streams of feedback (i.e., project documents review, open-ended survey, and an FGD), while sections four and five - discussion and conclusions – discuss and summarize the main points and outline steps for future research.

## An short recap of the conceptual framework

**Figure 2:**
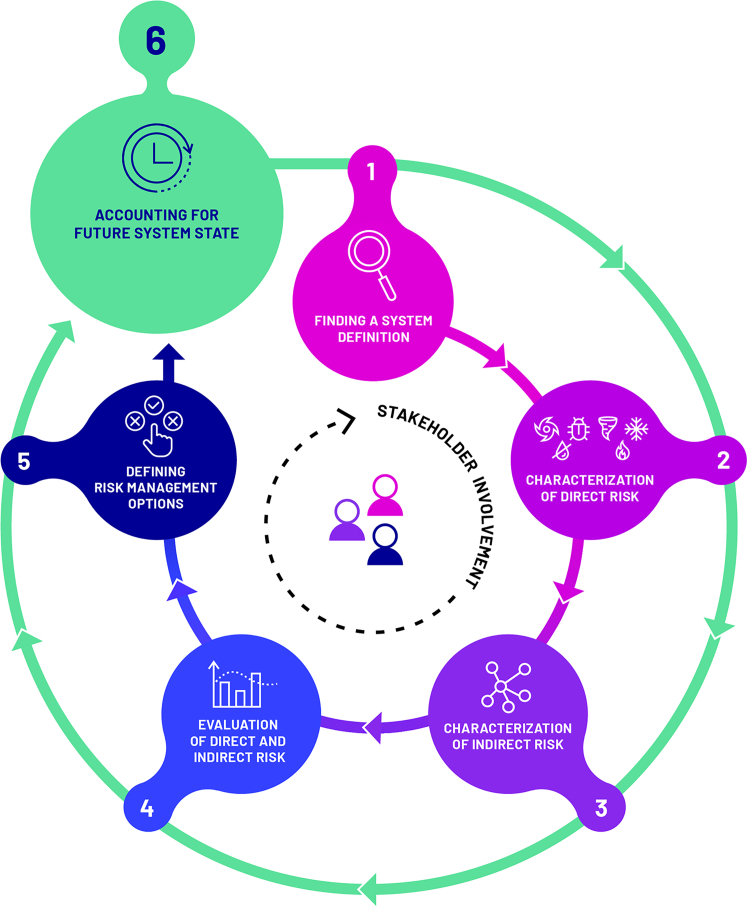
MYRIAD-EU framework for systemic multi-hazard and multi-risk assessment and management Source: Hochrainer-Stigler et al.^12^

The MYRIAD-EU framework is a structured, yet flexible, approach for assessing and managing systemic multi-hazard and multi-risk. A detailed description of the framework is available in the original paper by Hochrainer-Stigler et al.^12^ Here, we only present the main ideas underlying the framework and its six steps (Figure 2).

The framework was defined as “a frame one can work with” and did not prescribe specific tools, methods, and approaches for understanding risk assessment and management. Rather, it provided a broad framing capable of incorporating a variety of tools, methods, and approaches developed within MYRIAD-EU and beyond. As previously indicated, it was developed based on key insights from systemic risk research merged with research on multi-hazards and multi-risk. Systemic risk refers to risk that emerges from interdependencies between elements of a system, where failures or impacts can cascade and amplify, potentially affecting the system as a whole.^12^ Two fundamental principles from systemic risk theory underpin the framework: (1) the clear definition of the system—its boundaries and elements; and (2) the explicit mapping of dependencies (or interconnections) between system elements. The framework adopts a “system-of-systems” approach (i.e., understanding that each system is a subsystem of a wider system), which allows the necessary level of complexity with an explicit focus on dependencies. These dependencies can be hazard- or risk-related, and they allow for the integration of single, multiple, and systemic risks within one coherent framework. If no dependencies exist, multiple hazards may be analyzed independently, effectively as single risks; conversely, as interdependence increases, risks shift along a continuum from single risk to multi-risk to fully systemic risk. Building on these insights, the framework presents a six-step process for analyzing and managing risks across that spectrum, from single to multi- and systemic risk. The framework further builds on the existing multi-hazard and disaster risk management framework and follows the well-established procedures of the risk assessment process according to ISO 31010 norms, as explained in detail by Hochrainer-Stigler et al.^12^

The steps of the framework are the following (see Table 1 for an overview).

• *Step 1* includes finding a system definition that establishes clear system boundaries (e.g., based on exposure to different multi-hazards, administrative boundaries, or policy setting) and identifies the system’s constituent elements and their interconnections (e.g., through systems mapping exercises).
• Within *Step 2*, the framework focuses explicitly on direct risk; in other words, risk arising from direct contact between exposed, vulnerable elements and a hazard (e.g., earthquake) or a multi-hazard event (e.g., consecutive floods and droughts).^12^
• *Step 3* characterizes indirect risks, which refer to risks and losses that unfold through system interdependencies once direct risk has materialized.^12^
• Within *Step 4*, there is an evaluation process of direct and indirect risks, envisioned as a co-production exercise based on stakeholder and policy priorities and focusing on the determination of which risks need to be managed and which are acceptable, directly informing the decision-making process.
• Afterward, in *Step 5*, risk management options are selected according to the risk evaluation, with a special emphasis on risk management options that work across the scale of interrelated hazards.
• *Step 6* considers how the system under consideration might shift under global changes (e.g., climate and population change) and the risk management options introduced in Step 5. The framework is envisioned as an iterative process, as the system under consideration can evolve; for instance, risk management options can have implications for the realization of direct and indirect risks.

**Table 1:** The six-step process of the MYRIAD-EU framework

Step	Description
Step 1: System definition	Establish system boundaries and identify constituent elements and their interconnections (e.g., via systems mapping)
Step 2: Direct risk	Characterize risk arising from direct contact between exposed elements and hazard(s)
Step 3: Indirect risk	Capture indirect risks and losses via dependencies once direct risk has materialised
Step 4: Risk evaluation	Co-produce evaluation of risks (direct + indirect) with stakeholders to decide which risks to manage or accept
Step 5: Risk management options	Select and design risk management strategies that can address interrelated hazards across scales
Step 6: Future system states	Consider how the system might change (e.g., climate, population) and iterate risk management accordingly

It is important to note that the framework is not intended as a process for applying predescribed methods, tools, and approaches, as one cannot assume that a one-size-fits-all remedy exists in the form of a single approach; rather, one must adopt a toolbox-based approach that addresses different needs and can be useful on a case-by-case basis. Therefore, to support implementation of the framework in different contexts, the project developed guidance protocols accompanying each of the six steps, consisting of broad guiding questions and considerations (rather than fixed methods).^15^ These guidance protocols and the framework itself were iteratively refined based on pilot feedback from five case studies (Danube, North Sea, Scandinavia, Veneto, and Canary Islands) during the project period. During the project’s runtime (2021–2025), the MYRIAD-EU framework for systemic multi-hazard and multi-risk assessment and management and the accompanying guidance protocols were updated several times. The major steps in this development process, as well as the major changes made, are discussed in detail in Hochrainer-Stigler et al.^15^ The focus of what follows will be on the identified strengths, as well as limitations, regarding the implementation process as experienced by the MYRIAD-EU pilots.

## Critical reflections on the framework application in pilots

**Figure 3:**
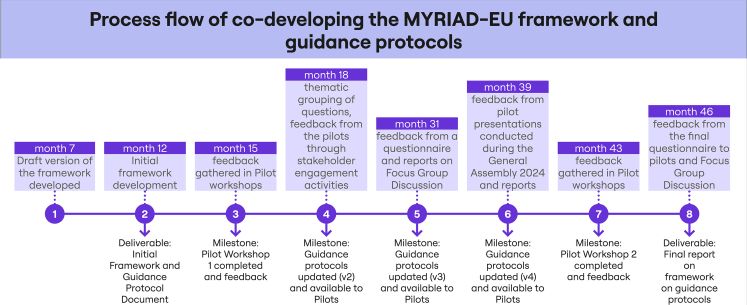
Timeline and major steps in the co-development process of the MYRIAD-EU framework for systemic multi-hazard and multi-risk assessment and management and the accompanying guidance protocols The timeline provides the project month when each development step took place and specifies whether it resulted in a project milestone document or a project deliverable.

Our analysis draws on three different data sources. First, a detailed review of relevant project deliverables was conducted to extract insights related to the implementation of the framework (see Figure 3, documents indicated as milestones [MS] and deliverables [D]). Second, an online survey (see S1 for questions) was used to examine key dimensions of the framework implementation as highlighted by each pilot lead and for each step of the framework application process. Finally, a joint FGD with pilot leads on the overall implementation process was held after the survey results had been analyzed. Taken together, these sources provide a multi-perspective view of the strengths, challenges, and lessons learned in applying the MYRIAD-EU framework across the five pilot regions. In what follows (and in Table 2), we present these strengths, challenges, and lessons learned grouped under the themes that emerged.

**Table 2:** Decision-relevant insights from applying the MYRIAD-EU framework across five pilots, reframing the critical reflections from the pilots and linking framework features to decision-making value, implementation requirements, and implications for uptake

Decision-relevant dimension	What the framework enables	Evidence from pilots	What is required in practice	Implications for decision-makers
Structuring complex decisions	breaks down multi-hazard, multi-sector risk into manageable steps while retaining a systemic view	all pilots emphasized the six-step structure as indispensable for organizing complexity and stakeholder dialogue	time for iteration; facilitation capacity; acceptance of non-linear processes	use the framework as a process guide, not a checklist; plan for iterative decision cycles
Understanding cascading and indirect risks	distinguishes direct hazard impacts from indirect, systemic effects propagating through interdependencies	Danube and Scandinavia pilots highlighted improved clarity on why non-exposed sectors experience impacts	data on interdependencies; qualitative system knowledge	supports anticipatory governance by revealing hidden vulnerabilities and spill-over effects
Supporting stakeholder dialogue	provides a shared reference point between technical and non-technical actors	Canary Islands and North Sea pilots reported improved workshop structuring and alignment of aims	translation into accessible language; tailored visuals and examples	treat the framework as a boundary object for co-production rather than a technical model
Enabling adaptive planning	encourages revisiting assumptions and revising decisions as new insights emerge	North Sea and Scandinavia pilots repeatedly looped back to earlier steps	explicit feedback loops; guidance that legitimises iteration	aligns with adaptive policy-making under deep uncertainty
Flexibility across contexts	allows tailoring to different spatial scales, governance settings, and data availability	Veneto valued multi-scale applicability; Danube highlighted freedom from predefined methods	clear minimum requirements; examples to avoid fragmentation	balance flexibility with light standardization to enable comparison and learning
Integrating tools and methods	offers a conceptual backbone to combine qualitative and quantitative tools	several pilots noted misalignment between framework steps and project tools	clear tool-step mapping; early integration of tools	enhances usability and avoids fragmented decision-support workflows
Institutional uptake and longevity	creates a coherent structure that can inform long-term DRM and adaptation strategies	Veneto stressed need for continued stakeholder engagement beyond project life	institutional anchoring; capacity building; policy alignment	framework adoption requires organisational commitment, not one-off application

### General perceptions of the framework

Across pilots, one of the strongest insights was that the framework provided an indispensable way to order and structure complexity. Beyond the technicalities of individual steps, the six-step process was seen as a necessary roadmap: it allowed pilots to break down highly interconnected problems into a sequence of manageable stages, while retaining a systemic perspective. This ordering function proved especially valuable for facilitating dialogue with stakeholders, as the framework acted as a shared reference point that helped bridge communication between technical and non-technical audiences. In pilots, workshops and FGs with key stakeholder groups would not have been feasible without the framework’s ability to organize and communicate multi-hazard dynamic risk complexities in an accessible way.

Another cross-cutting contribution concerns the framework’s explicit distinction between direct and indirect risks: direct risk refers to the immediate interaction between hazard(s) and exposed, vulnerable system elements, whereas indirect risk arises via interdependencies in the system once direct risk has materialized.^12^ While much of the disaster risk literature tends to treat any sectoral impact as a direct consequence, the framework narrows the definition of direct risk to the immediate interaction between hazard and exposed elements. Indirect risks are then defined as those that propagate through system dependencies once direct risks have materialized. This clarification introduces analytical rigor, improves comparability across contexts, and makes it easier for stakeholders to grasp why certain sectors experience impacts even when they are not directly exposed. By explicitly ordering risks in this way, the framework advances existing approaches that often conflate chains of impacts with direct consequences.

### Complexity and comprehensiveness

Across the different pilots and over the years, the framework’s complexity was one of the most frequently noted challenges. Especially in its initial versions, pilot leads had to have multiple interactions with the framework development team to fully understand the underlying concepts of the framework and how it could be implemented. For example, Step 1, “Finding a System Definition,” proved difficult to implement in the Canary Islands pilot, which described the step as “*highly ambiguous*” because it requires accounting for multiple factors, such as identifying relevant multi-hazards, setting analysis goals, reconciling potentially conflicting sectoral objectives, and examining the governance landscape. According to this pilot, the example provided in the guidance protocols was too specific to be transferable. The Danube pilot emphasized that defining the system required conscious decisions about what to exclude from the analysis and that drawing system boundaries, including geographical focus, as well as the choice of hazards and hazard interrelationships, is not a straightforward step in a large regional pilot covering 14 countries in the region. The issue of complexity in defining a system emerged in both large spatial pilots (Danube Pilot) and smaller ones (Canary Islands). The consistency of applying the framework across the five pilots was sometimes challenging, since each case study defined its system boundaries in different ways. We see this as a natural result of a framework designed to guide rather than prescribe how system boundaries should be drawn. However, this flexibility also highlights a key tension: while it allows pilots to adapt the framework to their specific contexts, it simultaneously complicates cross-pilot comparability and limits the potential for a more standardized approach to system definition.

Although the complexity was noted as a challenge, the framework was simultaneously appreciated for its comprehensiveness, as also highlighted by requests for a simplified elaboration of the framework. This approach was also valued because, despite the challenges of addressing such complexity, both scientists and decision-makers recognized the necessity of tools capable of adequately representing real-world conditions, that is, tools designed to operate within complex situations and processes. The framework’s attention to multi-risk analysis, systemic perspectives, and the distinction between direct and indirect effects, as well as accounting for transboundary impacts, was found to be useful for problem characterization and analysis. Pilots particularly appreciated how the six-step structure helped them “*break down the very complexity of multi-hazard risk into manageable stages*” (Scandinavia pilot). Some pilots, such as Veneto, valued that the framework operated across different spatial scales. In their case, the regional-scale application of the framework allowed them to address multiple interdependent issues simultaneously. The pilots reported that while the detailed implementation of each step (with metrics, methods, and precision) remains challenging and requires further methodological development, the framework and its iterative six-step process already provide a uniquely powerful way to structure complexity, enhance risk perception, and support dialogue.

### Structured process and iterative nature

The stepwise structure of the framework was praised for providing a clear and iterative process to assess multi-hazard risks. Thus, despite the perceived complexity of the framework itself, pilots emphasized how the steps help break down the complexity of multi-hazard and multi-risk analysis. For instance, the North Sea pilot noted that they frequently revisited previous steps in an evolving process. They also mentioned that the framework helped “*structure stakeholder inputs and formulate findings*,” while the Scandinavia pilot emphasized how it helped them “*understand the multiple aspects you have to consider when working on climate risks.*” This structured approach enabled pilots to engage with complex issues systematically. Pilots also underscored the iterative and non-linear nature of the process. As pilots advanced, they frequently revisited earlier steps to revise assumptions or adjust inputs based on new insights or data. Importantly, this was done in a structured manner, using the guidance protocols: they followed built-in “feedback loops” and prompting questions embedded in the protocols, which helped them systematically re-examine previous steps (e.g., redefining system boundaries, re-evaluating risk metrics) rather than doing so in an ad-hoc way. This structured revisiting was widely appreciated by pilot teams, as it helped them manage complexity without losing coherence.

### Holistic understanding of the DRM process and systems thinking

An especially strong aspect of the framework, according to the pilots, was its support for a holistic understanding of the disaster risk management process and systems thinking. The pilots valued how it encouraged them to think in interconnected ways about hazards, risks, and different sectors. For instance, the Scandinavia pilot used the framework to identify that water-related risks were central to their context and to clarify whether national- or local-level stakeholders were more appropriate to engage. Similarly, the Veneto pilot found the framework helpful in developing a conceptual model that tied together diverse risk analyses (e.g., multi-hazard risk analysis and disaster risk management pathways), enabling a more comprehensive understanding of disaster risk management in the Veneto Region.

### Stakeholder engagement and communication

A recurring theme across pilots was the challenge of communicating the framework to stakeholders. While it facilitated dialogue in some contexts, its conceptual complexity sometimes hindered understanding. The Canary Islands pilot reported difficulties in conveying abstract elements, whereas the North Sea and Veneto pilots found the framework helpful for structuring workshops and aligning aims. In the meantime, pilots emphasized that the framework facilitated more effective engagement with stakeholders, a benefit particularly emphasized by the Canary Islands and North Sea pilots. In the Canary Islands pilot, for instance, introducing the framework to stakeholders during the initial workshop had a dual function: it helped stakeholders understand the challenges, and it forced the pilots to internalize the framework’s structure more deeply. They referred to the framework as a “*shared reference point*,” which helped bridge communication between technical and non-technical audiences. This effect was echoed by the North Sea pilot, which appreciated the framework’s role in guiding stakeholder dialogues (i.e., it helped structure stakeholder workshops and align workshop aims).

Nonetheless, pilots noted that stakeholder engagement, and especially continued stakeholder participation throughout the project, required considerable effort. Translating concepts into accessible language, providing illustrative examples, and using visuals were essential. Therefore, aesthetics and accessibility played a significant role in stakeholder interaction: while the guidance protocols were generally seen as helpful, stakeholders, particularly in the Scandinavia pilot, found them to be dense in information and suggested they could benefit from more engaging visuals. Suggestions included incorporating real-life cases, example answers to guiding questions, and developing simple presentations introducing the framework to support understanding and use in practice. Finally, pilots recommended involving a broader range of stakeholders, including responders and practitioners. Some pilots (Veneto and Danube) emphasized that while the concepts were clear and the implementation feasible, a great deal of time was spent translating the material for stakeholder understanding, indicating a need for clearer language and illustrative examples of how specific steps could be implemented.

### Flexibility and adaptability

The framework’s non-prescriptive design was widely appreciated. Pilots valued the ability to adapt steps to regional contexts, available data, and pilot-specific challenges. For instance, the Danube pilot highlighted that the framework “*does not require a user to focus on a pre-determined set of methodologies,*” which allowed to experiment and iterate. This adaptability was especially appreciated given the complexity of the contexts in which pilots operated (e.g., the 14 countries covered by the Danube pilot). It allowed each pilot to interpret the framework in a way that matched its setting, including data availability and pilot-specific challenges. Although pilots acknowledged that the flexibility of the framework was one of its strengths, this adaptability also introduced complexity, as the iterative and non-linear nature of the framework implementation was identified as a key issue. As pilots advanced through the process of implementation, they frequently found themselves having to revisit earlier steps to revise their understanding or adjust inputs. As discussed, this occurred in a structured way, which was appreciated as helpful in managing complexity.

### Challenges and barriers

A significant cross-cutting barrier was the misalignment between the framework and the tools developed in MYRIAD-EU. Although the framework is not intended to prescribe specific tools, pilots found that tools such as DAPP-MR,^14^ storylines,^16^ or the multi-risk assessment software developed within the project could have been more clearly linked to the framework’s six-step structure. This led to what the Canary Islands pilot called a “*fragmented experience*” and what the Veneto pilot referred to as “*moderate use*” of the framework. This disconnect possibly influenced implementation and highlighted the need for earlier integration and coordination across tools and processes. Pilots interpreted this misalignment primarily as an operational reality of a project in which the framework was designed early on, while the tools were developed throughout the project cycle. For the purposes of developing a final version of the guidance protocols, pilots, in the survey, asked for a detailed mapping of how the tools developed within MYRIAD-EU align with the different framework steps, which was then implemented.^15^

Acquiring appropriate data was also recognized as a cross-cutting challenge. Even pilots considered more “data-rich,” such as the Scandinavia pilot, reported struggling at times with what type of data was needed or where to find it (e.g., for indirect risk assessment, where the Scandinavia pilot used a computable general equilibrium model, GRACE^17^). Another challenge concerned the background and capacities of those implementing or using the framework. For instance, physical scientists reported limited experience with qualitative methods such as interviews and workshops. In this sense, the framework’s interdisciplinarity was regarded as both an asset and a capacity burden, as it required expertise beyond usual disciplinary boundaries. Similarly, the background of stakeholders implementing the framework is highly relevant: pilots reasoned that in cases where stakeholders had strong technical backgrounds and understanding of disaster and climate risk, the process of implementing the framework might have been smoother, at least in the very beginning.

### Institutionalization and long-term uptake

A consensus emerged that effective institutionalization of the framework demands support mechanisms extending beyond typical research project cycles. For example, the Veneto pilot argued that stakeholder involvement must be facilitated in the long term if outputs such as the framework are to inform policy and regulatory processes. More generally, pilots emphasized that institutionalization requires ongoing support mechanisms, capacity building, and alignment with decision-making contexts.

### Suggestions for further development

Pilots offered several concrete suggestions to enhance framework usability, including:

• Developing a tiered engagement model with the framework (basic, intermediate, advanced), allowing users with different capacities and prior knowledge to engage at an appropriate level. The basic tier could rely on qualitative approaches, the intermediate tier on semi-quantitative methods, and the advanced tier on full quantitative analysis (e.g., disaster risk management pathways modeling).
• Embedding explicit prompts and “feedback-loop cues” throughout the guidance protocols to reinforce the iterative nature of the framework. Pilots noted that although the framework is designed to be iterative, this logic is not sufficiently reiterated in the current materials.
• Providing a detailed mapping of MYRIAD-EU tools and methods to each framework step, clarifying how tools such as DAPP-MR, storylines, and the multi-risk assessment software can be integrated into the six-step structure. This mapping has been partially implemented in later revisions but should be strengthened further.
• Enhancing visual communication materials, including simplified presentations, improved diagrams, real-world case examples, and sample answers to guiding questions. Pilots emphasized that clearer visuals and communication aids would improve stakeholder understanding and support practical implementation.
• Expanding guidance on stakeholder engagement, especially for involving responders, practitioners, and diverse local actors. Pilots noted that broader and more inclusive stakeholder participation would improve the practical relevance of the framework.
• Classifying suggested improvements into thematic categories, such as accessibility, methodological clarity, tool integration, and user-capacity alignment. Several points raised earlier in the Discussion already point toward potential enhancements, and grouping them would provide a clearer roadmap for future framework revisions.
• Providing clearer differentiation between “conceptual” and “operational” challenges would help future revisions. Several pilot comments mix conceptual ambiguity (e.g., unclear definition of systemic risk) with operational barriers (e.g., missing data or tools). Separating these would allow more targeted solutions.
• Adding more pilot examples as templates (e.g., short vignettes illustrating how specific steps were implemented in the Danube, Veneto, or Canary Islands cases) would support replication across contexts.
• Introducing a lightweight version of the framework for pilots with limited resources or narrow policy questions could address concerns that the full framework appears too comprehensive for many real-world decision contexts.

## Discussion

The increasing frequency, intensity, and compound nature of climate-related hazards underscore the urgency of systemic multi-hazard risk assessment. Climate change exacerbates existing vulnerabilities, creating new interdependencies and cascading effects that make traditional single-hazard approaches insufficient.^18^ Integrating such considerations highlights the relevance of the MYRIAD-EU framework not only for past or current risk management but also for anticipatory climate adaptation planning.

In a recent report, the EU Joint Research Center identified 47 risks, both natural and human-induced, that Europe is facing and which pose threats to people, infrastructure, and economies.^19^ The report specifically called for a systemic and multi-hazard approach to risk reduction and management, underlying the need for upscaling the application of the MYRIAD-EU framework. Beyond the pilot applications, insights presented in this paper point to broader implications for DRR research and practice. The framework’s iterative and systemic orientation offers a structured pathway for integrating emerging data, evolving stakeholder priorities, and new scientific findings—making it suitable for research contexts where risk landscapes shift rapidly and for operational settings where authorities must routinely update risk profiles. Highlighting these functions explicitly can support wider uptake beyond the project’s case studies. In particular, the framework’s iterative and systemic approach can support decision-making under climate change by helping stakeholders identify and prioritize risks that are emerging or likely to intensify in the future.

One of the strongest messages for future users from the pilots, who tested the framework in practice, is to embrace its iterative nature. For instance, both the Scandinavia and the Danube pilots emphasized that the process would involve revisiting earlier steps multiple times and that this should not be viewed as a failure of planning but rather as a strength of the approach. This iterative dynamic allows for the integration of new insights, shifts in understanding, and adaptations as the process unfolds. Moreover, climate change adaptation decision-making is directly supported by the framework, as the iterative six-step process allows practitioners to incorporate changing hazard conditions, long-term climate projections, and evolving vulnerability patterns into planning and risk reduction strategies. This is consistent with calls in the literature for iterative knowledge co-production across sub-systems,^20^ which enables both adaptive learning and progressively more sophisticated understandings of interdependencies.^21^

Alongside this, the Danube pilot advised users to be open-minded and experimental when selecting tools and methods. Instead of rigidly choosing a method at the outset, teams should stay flexible and allow the needs of each step and the evolving understanding of the system to inform the choice of approaches. This aligns with adaptive planning approaches,^22^^,^^23^ where flexibility is a strength but requires explicit guidance to avoid confusion. While several existing multi-hazard assessment frameworks (e.g., United Nations Office for Disaster Risk Reduction’s guidance on multi-hazard risk assessment, the INFORM Risk methodology, and other EU-funded initiatives such as PARATUS and MATRIX) emphasize the integration of multiple hazards, they often lack an explicit operationalization of systemic risk. What distinguishes the MYRIAD-EU framework is its system-of-systems perspective, the explicit separation of direct and indirect risks, and its emphasis on iterative co-production, which together provide a more comprehensive pathway for analyzing cascading and cross-sectoral impacts.

The framework’s value as a co-development tool was highlighted across pilots, bridging knowledge and perspectives across sectors, governance levels, and disciplines. The North Sea pilot pointed out that successful framework implementation also depends on the readiness and awareness of the users themselves. The Veneto pilot emphasized that teams should combine qualitative and quantitative tools to better capture the multifaceted nature of risk and that stakeholders should be engaged early and continuously. They noted that involving relevant stakeholders from the beginning through to the end of the process can enhance the legitimacy of the framework, help ground the analysis in local realities, and support the translation of results into actionable strategies. These insights are not only relevant for the pilots but also signal how the framework can be used by DRR practitioners to move beyond traditional single-hazard assessments toward a more integrated understanding of systemic vulnerabilities. This suggests that, outside the project, the framework could serve as a boundary object that helps DRR agencies, civil protection authorities, and sectoral planners jointly explore cross-sectoral dependencies that are often overlooked in conventional assessments. It also confirms evidence from participatory risk assessment literature that legitimacy and uptake depend equally on both methodological soundness and communication strategies.^24^^,^^25^^,^^26^ In several pilots, the framework functioned as a catalyst for dialogue, helping identify new stakeholders and opening conversations about cascading and indirect risks that might otherwise have been overlooked.

Our analysis shows that a systemic perspective can initiate a process that becomes very complex. These results are in line with recent calls for iterative knowledge co-production processes that need to be established across different sub-systems^20^ to increase understanding and an appreciation of the different entry points for managing risks within a systemic perspective.^27^^,^^28^ This also includes recognizing progress over time through various re-iterations and increasingly sophisticated understanding of the interactions and dependencies involved between stakeholders (for an application, see refs^27^^,^^29^; with regard to future risks, see the DAPP-MR approach^14^^,^^30^). In transdisciplinary contexts, such as those involving modeling and governance-related issues,^31^) and in settings where cross-boundary issues must be addressed, knowledge co-development can help make these complexities more manageable. It does so by improving communication and mutual learning about potential benefits and associated costs, as emphasized in multiple-dividends approaches.^9^^,^^32^

A major cross-cutting theme was the need to strengthen the guidance protocols, particularly by including more context-specific examples based on pilots, as suggested by the Canary Islands and Danube pilots. The Canary Islands pilot noted that having worked examples would have helped clarify the application of each step, while the Danube pilot saw this as a key area where the guidance could evolve, even if the framework’s structure remained unchanged. This reflects a broader lesson: technical guidance alone is insufficient. Usability can be enhanced through practical materials such as visualizations, case studies, and tailored communication tools. Such additions can help ensure that the framework is accessible not only to technical experts but also to policymakers and practitioners.

Overall, the feedback indicated that the framework itself does not require substantial improvements in terms of structure. The six-step structure was highly appreciated, and it was also emphasized that the framework has already gained traction in the wider scientific community (i.e., through the citation score of the Hochrainer-Stigler et al.^12^ paper). A clear, stepwise procedure has been praised as very helpful in breaking down a complex phenomenon into a set of manageable steps, while the framework maintained a holistic approach to identifying and tackling multi-hazard and systemic risks. The reduction of complexity through iterative knowledge co-development processes, as suggested above, requires not only appreciation but also the integration of solution-oriented structures that can facilitate navigation through complex problems in future applications. Yet, flexibility also came with ambiguity. Pilots valued the adaptability of the framework to their diverse contexts but noted that this reduced comparability across cases. This tension between flexibility and consistency is common in transdisciplinary frameworks.^33^ A potential way forward is a somewhat nested structure, where minimum requirements ensure comparability while optional modules allow for context-specific tailoring. Compared with other frameworks that emphasize standardized metrics or hazard-specific modeling, MYRIAD-EU provides a more adaptive structure that accommodates heterogeneous data environments and governance settings, which may make it particularly valuable for DRR agencies working across fragmented institutional landscapes.

In this regard, it must be noted that complexity as a research topic is not new and has been addressed by various disciplines (see the continuously updated Map of Complexity Sciences^34^). Indeed, there is increasing interest in the topic of complexity within disaster and climate change research,^35^ especially the emphasis on emergence, which positions research on complex systems in contrast to reductionist approaches. For instance, over the last decade, there have been growing advances in the climate adaptation field through the use of DAPP to develop a solution space and aid decision-making under deep uncertainty.^30^^,^^36^^,^^37^ Nevertheless, in many cases, the operational aspects needed to address complex problems and the practical pathways forward are seldom articulated, including within the disaster and climate change domains.^38^^,^^39^^,^^40^^,^^41^ Hence, we call for a “solution-oriented” approach, as not all problems need to be complex. The level of complexity required depends on the context, including the system scale and boundaries, as well as which interactions must be incorporated and which can be ignored. As is the case within systemic risk, the very nature of complexity seems to naturally call for multiple and plural perspectives. Indeed, some problems (e.g., simple, complicated, or complex ones) may require quantitative approaches, while others can be addressed through qualitative analysis. Furthermore, some are more related to risk governance processes, while others concern hard risk reduction measures (e.g., building dykes). The appreciation of multiple entry points to the problem, as well as different methodologies that can be used (alone or jointly), should be one of the cornerstones of such analyses, as is the case within systemic risk research (see Renn et al.^42^). A focus on communication between systems, particularly through visualization and knowledge co-production, offers novel and practical pathways for improving the management of complex problems in the future. Importantly, the framework’s utility extends to climate change adaptation contexts. By systematically incorporating multiple hazards, direct and indirect risks, and cross-sectoral interdependencies, it provides decision-makers with a structured approach to plan adaptive measures under uncertain and evolving climate conditions. This highlights its potential as a practical tool not only for DRR but also for supporting adaptation policies and long-term resilience strategies in climate-sensitive regions.

Finally, the pilots stressed that frameworks developed within research projects must be institutionalized to have a lasting impact. Without sustained support, they risk fading after the project cycle. This echoes earlier findings in disaster governance, which show that long-term partnerships with decision-making bodies and integration into regulatory structures are crucial for uptake.^26^^,^^43^ For MYRIAD-EU, this suggests that, beyond technical refinements, attention must be given to building communities, training materials, and institutional relationships that can sustain the framework’s use over time. In sum, the framework offers not only a research contribution but also a practical architecture for future DRR policy, complementing and extending existing multi-hazard approaches by embedding systemic thinking, iterative learning, and cross-sectoral coordination into risk assessment practice.

## Conclusions

In this paper, we present a critical reflection on the realities of implementing a conceptual framework for systemic multi-hazard and multi-risk assessment and management, as proposed by Hochrainer-Stigler et al.^2^ This framework was implemented over a 4-year period in five different pilot regions in Europe. Based on engagement with pilot teams through the analysis of multiple data sources (i.e., review of project reports, an open-ended survey, and a FGD), we identified a number of perceived benefits and challenges in the implementation, as well as advice for future users and for upscaling framework implementation. Within the framework development, we argued that a systemic perspective is beneficial for the assessment and management of multi-hazards and multi-risks. We emphasized that setting clear system boundaries, enabling a system-of-systems approach, and focusing on dependencies between system elements (either hazard- or sector-wise) provides a practical pathway for addressing multi-(hazard) risks within a step-by-step approach, thereby offering a frame one can work with.

From the onset of the project, it was evident that complexity increased substantially when the need to account for multi-hazard and multi-risk situations was introduced. In addition to data and modeling challenges, it was found that ongoing interactions with stakeholders are very resource-intensive and that making the results useful, usable, and used by them requires considerable effort. This is largely because the complexities involved must be reduced to manageable levels—both in terms of modeling, where modelers need to simplify systems enough to build accurate and computationally feasible models, and in terms of stakeholder engagement, where complexity must be broken down to ensure stakeholders can understand the problems at hand without being overwhelmed. The pilots highlighted that such simplification does not weaken the systemic perspective but enables meaningful dialogue, knowledge co-production, and ultimately, actionable outcomes.

Looking ahead, the multitude of large-scale hazard events, conflicts, and emergent risks, including pandemics and technological uncertainties, calls for an even greater appreciation of multi-risk contexts and a systemic perspective,^44^^,^^45^ as explicitly outlined in the European context.^19^ How such events unfold and influence disaster- and climate-related dimensions is a research topic not yet fully tackled, and it deserves more attention in the future. Current suggestions, such as the triple dividend or multiple dividend approaches,^32^^,^^46^ aim to link the disaster and climate risk domain with other systems, thereby providing a new way forward for holistically assessing different kinds of risks that are inherently interrelated. Our findings suggest that the MYRIAD-EU framework can serve as a steppingstone toward greater integration across different hazards and risks. With the concept of dependency at its core, it offers a way to connect and analyze interactions not only between natural, biological, and technological hazards but also with broader societal dimensions, such as well-being and health.^47^ To increase usability, pilots recommended strengthening the guidance protocols with more examples, simplifying communication materials, and engaging stakeholders from the outset. These elements will be crucial if the framework is to be mainstreamed into decision-making and institutionalized across scales. Knowledge co-production processes, as well as complexity-related methods in conjunction with frameworks that can differentiate between different types of problems, either human- or modelling-related,^48^ may provide promising ways forward for appreciating and handling these emerging challenges. Ultimately, the value of the framework lies not only in providing structure but also in fostering iterative, solution-oriented, and participatory approaches that make systemic risk management both feasible and impactful.

## Acknowledgments

The work was done as part of the HORIZON 2020 MYRIAD-EU Project, and the authors acknowledge funding from the 10.13039/501100007601European Union’s Horizon 2020 research and innovation program, call H2020-LC-CLA-2018-2019-2020, under grant agreement no. 101003276. M.C.d.R. also received funding through the 10.13039/501100003246Netherlands Organisation for Scientific Research (VENI; grant no. VI.Veni.222.169), while S.G.G. acknowledges funding from 10.13039/501100007757ACIISI, the Canary Islands Agency for Research. M.J.D. and R.C. publish with the permission of the Executive Director of the British Geological Survey (UK Research and Innovation, UKRI).

## Author contributions

Conceptualization, S.H.-S., R.S.T., and K.R.; investigation, S.H.-S., R.S.T., and K.R.; formal analysis, S.H.-S., R.S.T., and K.R.; methodology, S.H.-S., R.S.T., K.R., S.G., and R.C.; writing-original draft, S.H.-S., R.S.T., and K.R.; writing-review and editing, all authors; supervision, S.H.-S. and P.J.W.

## Declaration of interests

The authors declare no competing interests.

## Declaration of generative AI and AI-assisted technologies in the writing process

During the preparation of this work, the authors used ChatGPT-4 in order to improve the readability and language of the manuscript. After using this tool/service, the authors reviewed and edited the content as needed and take full responsibility for the content of the published article.

## Supplemental information

Document S1. Survey questions for the pilot teams
