# Supplementary material for: Operationalizing systemic multi-hazard and multi-risk assessment: Lessons from the MYRIAD-EU framework
Source: iScience. 2026 Feb 7;29(3):114935. doi: 10.1016/j.isci.2026.114935 (PMC12961295; doi:10.1016/j.isci.2026.114935)
Supplement: Document S1. Survey questions for the pilot teams [file mmc1.pdf]

iScience, Volume *29*

Supplemental information

Operationalizing systemic multi-hazard and multi-risk assessment: Lessons from the MYRIAD-EU framework

Stefan Hochrainer-Stigler, Robert Šakić Trogrlić, Karina Reiter, Anne Sophie Daloz, David Geurts, Lin Ma, Noemi Padrón-Fumero, Sharon Tatman, Silvia Torresan, Carmen D. Álvarez-Albelo, Veronica Casartelli, Roxana Ciurean, Maria Katherina Dal Barco, Jaime Díaz-Pacheco, Juan José Díaz-Hernández, Pedro Dorta Antequera, Melanie J. Duncan, Davide Mauro Ferrario, Sara García-González, Stefania Gottardo, Raúl Hernández-Martín, Abel López-Díez, David Romero Manrique, Diep Ngoc Nguyen, Marleen C. de Ruiter, Nikita Strelkovskii, and Philip J. Ward

iScience, Volume ■ ■

Supplemental information

Operationalizing systemic multi-hazard and multi-risk assessment: Lessons from the MYRIAD-EU framework

Stefan Hochrainer-Stigler, Robert Šakić Trogrlić, Karina Reiter, Anne Sophie Daloz, David Geurts, Lin Ma, Noemi Padrón-Fumero, Sharon Tatman, Silvia Torresan, Carmen D. Álvarez-Albelo, Veronica Casartelli, Roxana Ciurean, Maria Katherina Dal Barco, Jaime Díaz-Pacheco, Juan José Díaz-Hernández, Pedro Dorta Antequera, Melanie J. Duncan, Davide Mauro Ferrario, Sara García-González, Stefania Gottardo, Raúl Hernández-Martín, Abel López-Díez, David Romero Manrique, Diep Ngoc Nguyen, Marleen C. de Ruiter, Nikita Strelkovskii, and Philip J. Ward

SUPPLEMENTARY MATERIAL 1 (S1)

SURVEY QUESTIONS FOR THE PILOT TEAMS

Framework Update: Pilots Feedback

We kindly ask you to complete the survey linked below. The questions are grouped into three categories:

1. **Framework-specific questions:** reflections on your experiences with implementing the framework, including challenges encountered and lessons learned.
2. **Guidance protocol-specific questions:** feedback on the guidance protocol for each step, and a collection of tools, methods, and approaches used in your pilot.
3. **Framework co-development questions:** insights into how stakeholder knowledge was integrated into the framework development and stakeholder perceptions of the co-produced MYRIAD-EU framework.

BACKGROUND

1. Which Pilot are you involved in? *

- Danube
- North Sea
- Veneto
- Scandinavia
- Canary Islands

FRAMEWORK SPECIFIC QUESTIONS

1. How would you describe your overall experience implementing the framework in your pilot? Please reflect on both the benefits and the challenges you encountered.

____

2. To what extent did the framework guide the work in your pilot?

- Not at all
- To a small extent
- To a moderate extent
- To a great extent
- To a very great extent

3. Please briefly explain your answer to the previous question.

____

4. What are the key insights from your pilot implementation that could inform the further development and refinement of the framework? *In other words, what recommendations would you make regarding its theoretical foundations or the selection and design of steps (1–6)?*

____

5. Which steps of the framework did you find most challenging to implement, and why? *Please elaborate.*

6. Which steps of the framework did you find easiest to implement, and why?
Please elaborate.

7. Did the tools and methods developed in Work Packages 4, 5, and 6 support you in implementing the framework?

- o No, they did not help at all
- o They helped to a small extent
- o They were somewhat helpful
- o They were significantly helpful
- o They were essential for implementation

8. Please elaborate on your answer to the previous question. *For example, which specific tools or methods did you find most helpful, and why?*

9. What changes would you recommend for a revised version of the framework? *Please explain what changes you suggest, why they are needed, and how they could improve the framework.*

10. Based on your experience, what advice or recommendations would you give to future users of the framework?

GUIDANCE PROTOCOLS SPECIFIC QUESTIONS

In the section below, you will be asked to provide feedback on the guidance protocols for a specific step of the framework. Your feedback may include both strengths and limitations of the current guidance (or indicate if no changes are needed), as well as suggestions for improvement. You are also welcome to propose additional guiding questions or suggestions that could help users better navigate the step. Importantly, we ask you to share concrete examples of tools, methods, or approaches you used to implement this step. Please note that detailed descriptions are not necessary—we are aiming to map and collect examples from the pilots in order to enrich the existing guidance protocols with practical insights.

1. To what extent have you been using the guidance protocols?

- o I have not used the guidance protocols at all
- o I have used them to a small extent
- o I have used them occasionally
- o I have used them regularly
- o I have relied on them extensively

2. Please shortly elaborate on your answer to the previous question. For instance, why you did not use it, what parts of the protocol did you use the most and why, etc..

STEP 1: FINDING A SYSTEM DEFINITION

1. Please provide feedback on the guidance you have received for Step 1?

____

2. Please provide concrete examples of tools, methods, and approaches you have used in your pilot for operationalizing Step 1 that you think should be included in the final guidance protocol. *

____

STEP 2: DIRECT RISK

1. Please provide feedback on the guidance you have received for Step 2? *

____

2. Please provide concrete examples of tools, methods, and approaches you have used in your pilot for operationalizing Step 2 that you think should be included in the final guidance protocol. *

____

STEP 3: INDIRECT RISK

1. Please provide feedback on the guidance you have received for Step 3? *

____

2. Please provide concrete examples of tools, methods, and approaches you have used in your pilot for operationalizing Step 3 that you think should be included in the final guidance protocol. *

____

STEP 4: EVALUATION OF DIRECT AND INDIRECT RISK

1. Please provide feedback on the guidance you have received for Step 4?

____

2. Please provide concrete examples of tools, methods, and approaches you have used in your pilot for operationalizing Step 4 that you think should be included in the final guidance protocol.

____

STEP 5: IDENTIFYING RISK MANAGEMENT OPTIONS

1. Please provide feedback on the guidance you have received for Step 5? *

____

2. Please provide concrete examples of tools, methods, and approaches you have used in your pilot for operationalizing Step 5 that you think should be included in the final guidance protocol. *

____

STEP 6: ACCOUNTING FOR FUTURE SYSTEM STATE

1. Please provide feedback on the guidance you have received for Step 6?

____

2. Please provide concrete examples of tools, methods, and approaches you have used in your pilot for operationalizing Step 6 that you think should be included in the final guidance protocol. *

____

FRAMEWORK CO-DEVELOPOMENT SPECIFIC QUESTIONS

1. What factors have supported the successful integration of stakeholder knowledge into developing the MYRIAD-EU framework and guidance protocols? *Please identify specific factors (e.g., communication, alignment of goals, mutual understanding) and explain why they were important for success.*

____

2. What challenges did you encounter in facilitating the integration of stakeholder knowledge into the development of the MYRIAD-EU framework? How, if at all, did these challenges impact the progress of the Pilot? *Please describe any specific challenges and their effects.*

____

3. When testing and implementing the framework's steps or guidance protocols with stakeholders, were any adjustments made to the framework? *If so, please describe the adjustments and the reasons behind them (e.g., to align with local context or address stakeholder needs). If no adjustments were necessary, please indicate that as well.*

____

4. In your opinion, did the stakeholder engagement and co-development process increase the likelihood of successfully implementing the framework in practice or policy? *Please explain your answer, specifying whether the likelihood of success in practice, policy, or both increased due to the engagement process.*

____

5. In your opinion, how do stakeholders perceive the likelihood of the framework being adopted by practitioners, decision-makers, and policymakers in real-world settings? *For instance, did their feedback highlight any instances where policies or Disaster Risk Management actions will be influenced by the framework's findings? Please provide any relevant feedback or examples shared by stakeholders.*

____
